# Acceptance of digital health services among older adults: Findings on perceived usefulness, self-efficacy, privacy concerns, ICT knowledge, and support seeking

**DOI:** 10.3389/fpubh.2022.1073756

**Published:** 2022-12-13

**Authors:** Mario R. Jokisch, Laura I. Schmidt, Michael Doh

**Affiliations:** ^1^Institute of Gerontology, Heidelberg University, Heidelberg, Germany; ^2^Catholic University of Applied Sciences Freiburg, Freiburg, Germany; ^3^Institute of Psychology, Heidelberg University, Heidelberg, Germany

**Keywords:** ICT, public health, TAM, digital health, older adults, self-efficacy, support

## Abstract

**Background:**

Over the last decade, the rapid advancements in information and communication technologies (ICTs) have also driven the development of digital health services and applications. Older adults could particularly benefit from these technologies, but they still have less access to the Internet and less competence in using it. Based on the empirical literature on technology acceptance among older adults, this study examines the relations of perceived usefulness, self-efficacy, privacy concerns, ICT knowledge, and support seeking (family, informal, formal/institutional) with older adults' intention to adopt new digital health services.

**Methods:**

The study included 478 older adults who participated in an online or paper/pencil questionnaire (*M* = 70.1 years, SD = 7.8; 38% male). Sociodemographic characteristics, subjective health status, and variables related to technology acceptance were assessed.

**Results:**

Latent structural equation modeling revealed that higher perceived usefulness, higher self-efficacy regarding digital health technologies, and lower privacy concerns contributed to a higher intention to use digital health services among older adults. Contrary to our expectations, general ICT knowledge was not a significant predictor. Older adults who reported seeking more support regarding technology problems from family members and formal/institutional settings also reported higher usage intentions, whereas informal support was not as relevant. Furthermore, higher age was associated with higher perceived usefulness and lower self-efficacy.

**Discussion:**

Future studies should further explore mediating factors for intention and actual use of digital health services and develop educational programs including follow-up assessments.

## Introduction

Over the last decade, the rapid advancements in information and communication technologies (ICTs) have also driven the development of digital health services and applications. In a similar manner to the US, European national health care systems have started to include digital services, i.e., remote communication with health care providers, e-prescription, scheduling medical appointments online, redirecting to online portals for health information or education, and online personal health records (1, 2).

However, most digital health services are just in the phase of implementation and are not yet accessible to a large extent. For Germany, the “Act to Improve Healthcare Provision through Digitalization and Innovation” (Digital Healthcare Act—DVG) (3) was approved in November 2019 by the German Bundestag, but central services such as the electronic medical record, medication prescriptions, and sick notes for the employer were first introduced in 2021 or later (4).

Although persons of all ages are meant to profit from digital health solutions, older adults could particularly benefit: With the frequency and complexity of medical issues rising with age alongside potential mobility impairments, such solutions might empower older people to still manage their health actively and safely. However, access to those services delivered over digital platforms may represent a challenge to some older adults. Diffusion of ICTs is still ongoing, with older adults still being less likely to use the Internet and web-connected ICTs in comparison to the general population (5–7). Moreover, in 2021, 92% of people aged 60–70 were expected to have used the Internet, but only 51% of people over 80 were expected to have done so (8). On the one side, this shows that many older adults can draw on ICT skills and knowledge when it comes to adapting new digital health services, on the other side, it can be a challenge for those who have limited access to the internet and/or less previous experience. Besides access as a necessary precondition, little is known regarding the views and beliefs of older people regarding digital health services.

### Acceptance of digital (health) technologies among older adults

Although the usage of digital health applications might come with large potential for the individual as well as for public health strategies, the bottleneck might be a lack of acceptance among older adults. The technology acceptance model (TAM) represents one of the widely used frameworks and assumes that perceived usefulness and perceived ease of use are major predictors of intention to use a given technology (9). Studies addressing empirical evidence related to the TAM with old and very old adults are rare, as are studies with an explicit focus on digital health services (10). However, there is evidence that associations of the TAM change with increasing age (11). In the “young-old” age between 60 and 75 years, when many resources are still available for most individuals, perceived usefulness has been shown to be crucial. In more advanced age (75+), tech-related self-efficacy has seemed to gain importance, whereas ease of use has appeared to be less relevant. Technology-related self-efficacy is relevant for older adults, regardless of whether they have a high or low level of digital competence, and is positively associated with various technology-related biographical experiences (12). Correspondingly, an increasing body of research does not confirm the relevance of perceived ease of use among the older population [e.g., (13, 14)]. With regard to new digital health solutions, for which less experience is available, this points to the high importance of exploring respective self-efficacy beliefs that may represent future starting points for interventions.

Fewer studies based on the TAM are available regarding acceptance of digital health technologies among older adults. Zheng et al. (15) state that in addition to social support and computer self-efficacy, the search for health information can be a key motivator for Internet use among older adults. Chang and Im (16) showed that perceived usefulness and simplicity were important predictors of health information seeking. In addition, those older adults who did not search for health information on the Internet had lower computer self-efficacy (17). Harris and Rogers (18) conducted a qualitative study and interviewed older adults with chronic health conditions about their acceptance of health technologies. The TAM factors were confirmed and, in addition, technic-specific factors such as advice acceptance, compatibility, convenience, facilitating conditions, subjective norm, trust, and privacy issues were also relevant for acceptance. Also from a more general perspective on smart technologies, privacy concerns are among the most common barriers preventing older adults from using digital devices and services (19).

Only remotely related to the TAM, the role of digital competence as well as (social) support for the use of digital health services among older adults has been investigated recently. For example, in a representative population-based Finnish study (*N* > 4,400), high digital competence was able to mediate the age-related decline in online services use (i.e., receiving test results, renewing prescriptions, and scheduling appointments), but only up to around the age of 80 years (20). This can be seen as an indication that general ICT knowledge and skills are an important predictor for the acceptance of new digital health services. Regarding level of support, initial qualitative and mixed-method data from the US and Israel indicate positive associations of support by either family members or non-kin intergenerational mentoring regarding the use of online health services (21, 22).

### Research questions

Following these promising but still limited findings, our research aim was to test associations based on an extended TAM in order to predict the intention to use digital health services among older adults. In detail, and in accordance with the core of the TAM, we assume that perceived usefulness emerges as most important in predicting the intention to use digital health services. For self-efficacy beliefs tailored to digital health services, we hypothesize higher scores to be associated with a high level of intention. Additionally, we assume a positive relation with perceived usefulness, as people with high self-efficacy might recognize more opportunities to integrate digital health services in their lives. Regarding potential barriers, we expect that higher privacy concerns with respect to digital health services have a negative effect on the intention to use such digital applications. Regarding prior experience, we assume that older adults who report having higher knowledge about ICTs in general also have a higher intention to use digital health services.

For support, we hypothesize that older people who report higher support-seeking behavior also express higher intentions, perceived usefulness, and self-efficacy regarding digital health services. We exploratively aim to analyze if patterns differ with regard to support seeking among family members, voluntary/informal sources, or professional sources. On an exploratory level, we also wanted to investigate whether high ICT knowledge contributes to fewer privacy concerns and higher digital health self-efficacy.

## Design and methods

### Recruitment and sample

The study is part of a larger project, called “Healthy Aging in Baden-Wuerttemberg,” funded by the Ministry of Social Affairs and Integration Baden-Württemberg, which aimed to provide educational services with a focus on digital health services for older adults. In 2021, 256 events were provided for older people in which information and practice possibilities on digital health functions were offered (i.e., electronic patient record, electronic health card, digital services in pharmacy care, video contact with one's doctor, health apps). A total of 2,510 people attended the events, of whom 559 individuals participated in an online or paper/pencil questionnaire. Of these, 81 were excluded because they were under 50 years old, yielding a cohort of 478 older adults who were included in the study. On average, participants were 70.1 years old (SD = 7.8, age range: 50–91 years), more often female (63%), with a high degree of education (high 54%, medium 31%, low 15%), and mainly reporting a good to sufficient health status (*M* = 4.6, SD = 0.8) (Table 1).

**Table 1:** Descriptive statistics and correlations for study variables.

**Variable**	***M***	***SD***	**1**	**2**	**3**	**4**	**5**	**6**	**7**	**8**	**9**	**10**	**11**	**12**
1. Age	70.1	7.8	–											
2. Gender (male %)^a^	37.61		−0.02	–										
3. Education (high %)^b^	53.64		−0.25^***^	−0.15^**^	–									
4. Subjective health ^c^	4.6	0.8	−0.12^*^	−0.06	0.08	–								
5. Intention^d^	3.8	1.0	−0.05	0.10^*^	0.15^**^	−0.04	–							
6. Perceived usefulness^d^	3.6	0.9	0.06	0.04	0.03	−0.03	0.58^***^	–						
7. Self-efficacy	3.2	1.0	−0.16^**^	−0.02	0.09	0.09	0.31^***^	0.34^***^	–					
8. Privacy concerns^d^	3.0	0.9	0.03	0.00	0.07	−0.05	0.28^***^	0.25^***^	0.15^**^	–				
9. ICT knowledge^c^	4.0	1.0	−0.20^***^	−0.04	0.17^**^	0.22^***^	0.13^**^	0.12^*^	0.29^***^	0.23^***^	–			
10. Family support^d^	3.6	1.3	0.06	−0.05	0.04	0.15^**^	0.06	−0.03	−0.002	0.09	0.04	–		
11. Informal support^d^	3.1	1.2	0.13^*^	0.06	−0.13^*^	−0.01	−0.01	0.03	0.14^**^	−0.01	−0.07	−0.01	–	
12. Formal/institution support^d^	3.6	1.2	−0.001	0.06	−0.12^*^	−0.03	0.18^***^	0.26^***^	0.17^***^	0.07	0.11^*^	−0.05	0.31^***^	–

*N* = 478.

a Male = 0, women = 1.

b Education: low = 1, medium = 2, high = 3.

c 1 = “very bad” to 6 = “very good”.

d 1–5, higher scores indicate more positive scores.

* *p* < 0.05;

** *p* < 0.01;

*** *p* < 0.001.

### Measures

*Intention* to use digital health services (e.g., “assuming I had access to digital health services, I intend to use it”) and *perceived usefulness* (e.g., “using digital health services is useful for my life”) were measured with two items each, based on Davis et al. (9). Evaluations of *privacy concerns* were assessed *via* two items from the Tele-healthcare Satisfaction Questionnaire—Wearable Technology [e.g., “The storage or further processing of my personal health data may have negative consequences”; TSQ-WT, (23)]. To assess self-efficacy related to digital health services, two items based on the Short Scale for Measuring General Self-Efficacy Beliefs (24) were adopted (e.g., “If problems arise when using digital health services, I can solve them on my own”). *Support seeking* in the case of need for help with digital health services was assessed *via* three self-developed items asking for the level of agreement regarding support from (1) family members (e.g., “When I need help with digital health services, I seek it from family members”), (2) informal/voluntary sources (e.g., “…I seek it from volunteer peer support”), or formal/institutional sources (e.g., “…I seek it from information events, training courses”). All items mentioned above were answered on a five-point Likert scale ranging from 1 (do not agree at all) to 5 (fully agree). *General ICT knowledge* was measured by asking respondents to rate their knowledge regarding computers, smartphones, tablets, wearables, and the Internet on a scale from 1 (very poor) to 6 (very good). Participants' current health status was assessed using the same response scale. Internal consistency was good to excellent (intention: α = 0.76; perceived usefulness: α = 0.86; self-efficacy: α = 0.88; privacy concerns: α = 0.74; ICT knowledge: α = 0.83).

### Statistical analysis

Statistical analyses were performed using SPSS 28.0 for descriptive statistics and Amos 24.0 for latent structural equation modelling. We applied a latent structural equation model for the entire sample. Four latent factors were indicated by two items each (perceived usefulness, self-efficacy, privacy concerns) and one latent factor by five items (ICT knowledge). Support in the form of family members, informal/voluntary support services, and formal/institutional support services were included as a manifest variable with one item each. Sex, subjective health, age, and education were also entered as manifest variables with relations to perceived usefulness, self-efficacy, privacy concerns, support, and ICT knowledge.

Model fit was tested using the comparative fit index (CFI) and the root-mean-square error of approximation (RMSEA). A CFI score ≥ 0.90 and a RMSEA score ≤ 0.08 were interpreted as an acceptable model fit, while a CFI score ≥ 0.95 and a RMSEA score below ≤ 0.05 represented a good model fit (25). With regard to missing data treatment, full information maximum likelihood was applied (26).

## Results

Descriptive and correlational results are presented in Table 1. Mean scores of all items were settled around the average or in the positive range of the scales, with considerable variation.

### Latent structural equation modelling

**Figure 1:**
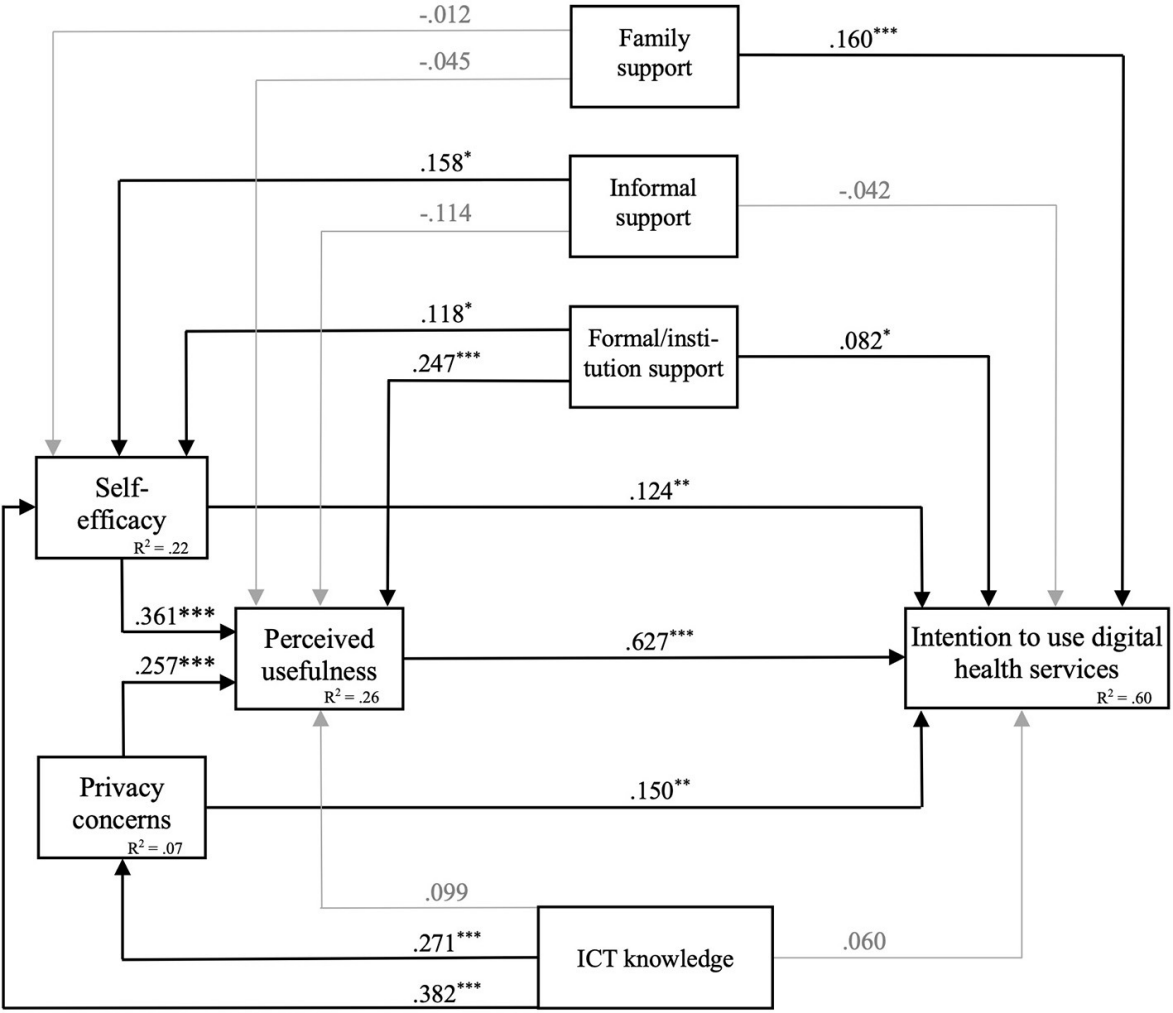
Path model predicting the intention to use digital health services. Significant β coefficients are depicted black. *R*^2^ = total variance explanation. ^*^p < 0.05; ^**^p < 0.01; ^***^p < 0.001.

The model including all study variables yielded good overall fit indices (CFI = 0.973; RMSEA = 0.036). Detailed results can be derived from Table 2, and the path model including β coefficients is depicted in Figure 1. The overall model explained 60% of the variance in intention to use digital health services. Additionally, 26% of the variance in perceived usefulness, 22% of the variance in self-efficacy and 7% in privacy concerns could be explained.

**Table 2:** Results of the study variables in the structural equation model for digital health services.

**Path**	***b***	**ß**	***SE***	***p***
Intention ← Perceived usefulness	0.666	0.627	0.053	<0.001
Intention ← Self-efficacy	0.131	0.124	0.049	0.008
Intention ← Privacy concerns	0.227	0.150	0.061	<0.001
Intention ← Family support	0.12	0.160	0.026	<0.001
Intention ← Informal support	−0.033	−0.042	0.031	0.286
Intention ← Formal/institution support	0.067	0.082	0.033	0.038
Intention ← ICT knowledge	0.068	0.060	0.054	0.209
Perceived usefulness ← Self-efficacy	0.359	0.361	0.059	<0.001
Perceived usefulness ← Privacy concerns	0.365	0.257	0.076	<0.001
Perceived usefulness ← Family support	−0.032	−0.045	0.034	0.342
Perceived usefulness ← Informal support	0.086	0.114	0.040	0.050
Perceived usefulness ← Formal/institution support	0.192	0.247	0.040	<0.001
Perceived usefulness ← ICT knowledge	0.106	0.099	0.070	0.129
Self-efficacy ← Family support	−0.009	−0.012	0.035	0.808
Self-efficacy ← Informal support	0.12	0.158	0.041	0.003
Self-efficacy ← Formal/institution support	0.092	0.118	0.041	0.025
Self-efficacy ← ICT knowledge	0.411	0.382	0.074	<0.001
Privacy concerns ← ICT knowledge	0.204	0.271	0.057	<0.001

N = 478; Total variance explanation in intention: 60%.

As assumed, perceived usefulness was positively related to the intention to use digital health services (β = 0.63, *p* < 0.001). This was also the case for health-related self-efficacy (β = 0.12, *p* = 0.008) and privacy concerns (β = 0.15, *p* < 0.001). In terms of support, older adults who reported seeking family support (β = 0.16, *p* < 0.001) and formal/institutional support (β = 0.08, *p* = 0.038) reported higher intention levels. Contrary to our assumption, informal support was not a significant predictor for the intention to use digital health services (β = −0.04, *p* = 0.286). Also contrary to our assumptions, general ICT knowledge was not related to the intention to use digital health services (β = 0.06, *p* = 0.209).

As hypothesized, there was a clear positive association between perceived usefulness and health-related self-efficacy in using digital health services (β = 0.36, *p* < 0.001). Similarly, fewer privacy data concerns contributed to higher perceived usefulness (β = 0.26, *p* = < 0.001). Regarding the role of support, older people who reported seeking more formal/institutional support exhibited higher scores in perceived usefulness (β = 0.25, *p* < 0.001). In addition, there was a marginal association between informal support seeking and perceived usefulness (β = 0.11, *p* = 0.050) and no relation with support from family members (β = −0.45, *p* = 0.342).

With regard to health-related self-efficacy, there was no connection with support seeking *via* family (β = −0.00, *p* = 0.974), but a association between informal support sources and health-related self-efficacy (β = 0.16, *p* = 0.003). Older adults who reported seeking significant formal/institutional support showed higher health-related self-efficacy (β = 0.18, *p* = 0.003).

Also contrary to our assumptions, general ICT knowledge was not related to the intention to use digital health services (β = 0.06, *p* = 0.209). The explorative analyzed relation between perceived usefulness and ICT knowledge could not be established (β = 0.10, *p* = 0.129). However, older adults with a high level of ICT knowledge reported a higher self-efficacy (β = 0.382, *p* = < 0.001) and less privacy concerns (β = 0.271, *p* = < 0.001).

Regarding the control variables, age showed no relation with either intention (β = 0.04, *p* = 0.296) or privacy concerns (β = 0.00, *p* = 0.116). However, age was positively related to perceived usefulness (β = 0.14, *p* = 0.005) and negatively related to health-related self-efficacy (β = −0.16, *p* = 0.003), indicating that participants who were older recognized higher perceived usefulness of digital health services but reported lower health-related self-efficacy to use them. Gender was positively associated with intention (β = 0.091, *p* = 0.007), with women reporting a higher intention to use digital health services. Higher education status contributed to a higher intention to use digital health services (β = 0.12, *p* = 0.002) but did not show significant relations otherwise. ICT knowledge also declined with increasing age (β = −0.27, *p* = 0.001) and was positively related to better health status (β = 0.20, *p* = 0.001). All results are depicted in Supplementary Table 1.

## Discussion

Our study aimed to explore older adults' perceptions regarding digital health services, in order to provide initial insights on associations within an extended TAM framework. In summary, higher perceived usefulness and self-efficacy, more perceived family and formal support, and low privacy concerns contributed to a higher intention to use digital health services, among our relatively well-educated and healthy sample of older adults.

First, these results show that established factors that predict technical acceptance in other technology areas such as digital health services are also relevant among older adults. Second, perceived usefulness was the dominant factor in the model, whereas health-related self-efficacy and privacy concerns were also significant but exhibited lower contributions to the intention to use digital health services. Age itself was not directly linked to usage intentions but was predictive of higher perceived usefulness, highlighting the importance that digital health services offers for the oldest age group. As older age was also associated with lower health-related self-efficacy beliefs, self-efficacy or related constructs such as perceived control should be investigated more deeply and longitudinally as potential mediators between age and acceptance with respect to digital health services and tools. Furthermore, it should be taken into account that if newly developed digital health services have lower or insufficient usability, factors such as ease of use might gain importance for the decision-making process.

Older adults with more ICT knowledge were found to have higher self-efficacy and reported less privacy concerns, but there was no relation to perceived usefulness and intention. This could be taken as a first indication that general ICT knowledge is not directly related to the decision to use a digital health service and that this technologies may be a separate issue from overall ICT adoption. However, the selectivity of the sample should be considered in this context. Our respondents were older adults with a relatively high level of education and above-average ICT knowledge, and can be classified as early adopters who engaged with ICT at an early stage (27). In a more diverse sample including less privileged older adults with insufficient ICT background skills, effects on acceptance might still be expected.

Our finding that women reported higher intentions to use digital health services might also relate to gender roles and social norms in general, as women tend to be the family caregiver, i.e., they were in charge of making health care decisions for children earlier in life and often take care of medical appointments for husbands or older relatives, whereas traditional masculine behavioral patterns can prevent men from further dealing with health-related services [i.e., (28, 29)]. However, more research is needed that also focuses on women with lower education levels as well as those from rural areas, as our sample was also biased in terms of having a large percentage of well-educated women in our survey.

### Limitations

Our study has some limitations that need to be acknowledged. First, as addressed above, our sample was selective with regard to mainly higher education levels as well as the fact that participants were recruited *via* events that relate to technology issues. Second, our cross-sectional results do not allow causal interpretation, and longitudinal research is needed that investigates actual adoption of digital health services at the time they are available for the public, which can be expected in the next year for some services (i.e., e-prescription). Third, our data collection took place during the COVID-19 pandemic, which might have (positively) influenced perceptions of digital health services that in part replace physical contact and thus protect against infection. Fourth, we used predominantly short scales and parsimonious measures that might profit from extensions in future studies. For example, to better understand the role of ICT knowledge and also competencies, we recommend using comprehensive questionnaires such as the Mobile Device Proficiency Questionnaire (30).

### Practical implications and outlook

This study provides initial indications regarding the type of support that is relevant for older adults when adopting new digital health services. In particular, formal education was rated as important and was associated with more favorable scores regarding self-efficacy, usage intention, and usefulness of digital health services. This indicates two points: first, formal education seems to be relevant for the decision to use new digital health services. It is therefore important to provide suitable educational offerings to accompany the introduction of new innovations in the health sector. Second, it can be assumed that for this highly educated sample, formal educational opportunities are marked by high visibility and easy availability. However, this might not be the case for people with a low level of education who do not make (continuous) use of these existing educational structures, or for older adults with (mild) cognitive impairment (31, 32).

In this context, non-formal education programs can play an important role. Voluntary programs can be tailored more closely to the needs of older people who do not feel addressed in traditional courses offered by formal education programs (33). Older adults who are representative of those surveyed here, who exhibit a high interest in new digital health services, have a high level of ICT knowledge, and recognize the benefits of technology, should be recruited as volunteers. This creates role models who have already overcome problems with technologies that inexperienced groups face. *Via* vicarious experience, role models offer a means to increase self-efficacy (34). Moreover, volunteering has numerous positive effects for those who engage in it. For example, older adults who were active in ICT-related contexts have been shown to gain more ICT knowledge, to experience increases in general self-efficacy, and to exhibit reduced feelings of obsolescence (35). As the field of digital health services is embedded in a dynamic innovative process of digital transformation within health care systems, more research is needed to determine preferences and identify facilitators or barriers among older adults. Educational programs should be carefully designed with older adults involved as active partners and should be tested using robust experimental and longitudinal designs.

## Data availability statement

The raw data supporting the conclusions of this article will be made available by the authors, without undue reservation.

## Author contributions

MJ: conceptualization, investigation, writing—original draft, methodology, and formal analysis. LS: conceptualization, validation, and writing—review and editing. MD: writing—review and editing, resources, and project administration. All authors contributed to the article and approved the submitted version.
