# Supplementary material for: Acceptance of digital health services among older adults: Findings on perceived usefulness, self-efficacy, privacy concerns, ICT knowledge, and support seeking
Source: Front Public Health. 2022 Dec 13;10:1073756. doi: 10.3389/fpubh.2022.1073756 (PMC9792847; doi:10.3389/fpubh.2022.1073756)
Supplement: Supplementary file 1 [file Table_1.docx]

| **Supplementary Table 1** | | | | | | |
| --- | --- | --- | --- | --- | --- | --- |
| *Results of the Control Variables in the Structural Equation Model for Digital Health Technologies* | | | | | | |
|  |  |  | *b* | *ß* | *SE* | *p* |
| Intention | 🡨 | Age | 0.005 | .040 | .005 | .296 |
| Intention | 🡨 | Subjective health | -0.01 | -.008 | .043 | .820 |
| Intention | 🡨 | Sex | 0.185 | .091 | .069 | .007 |
| Intention | 🡨 | Education | 0.154 | .116 | .049 | .002 |
| Perceived usefulness | 🡨 | Age | 0.016 | .137 | .006 | .005 |
| Perceived usefulness | 🡨 | Subjective health | -0.001 | -.001 | .054 | .989 |
| Perceived usefulness | 🡨 | Sex | 0.093 | .049 | .087 | .286 |
| Perceived usefulness | 🡨 | Education | 0.026 | .021 | .063 | .677 |
| Self-efficacy | 🡨 | Age | -0.018 | -.157 | .006 | .003 |
| Self-efficacy | 🡨 | Subjective health | -0.091 | -.085 | .055 | .098 |
| Self-efficacy | 🡨 | Sex | -0.062 | -.034 | .090 | .488 |
| Self-efficacy | 🡨 | Education | 0.070 | .058 | .064 | .276 |
| Privacy concerns | 🡨 | Age | 0.002 | .021 | .004 | .116 |
| Privacy concerns | 🡨 | Subjective health | -0.021 | -.027 | .040 | .602 |
| Privacy concerns | 🡨 | Sex | 0.051 | .039 | .066 | .445 |
| Privacy concerns | 🡨 | Education | 0.082 | .095 | .048 | .089 |
| ICT knowledge | 🡨 | Age | -0.025 | -.226 | 0.006 | .001 |
| ICT knowledge | 🡨 | Subjective health | 0.207 | .198 | 0.052 | .001 |
| ICT knowledge | 🡨 | Sex | 0.112 | .063 | 0.086 | .193 |
| ICT knowledge | 🡨 | Education | -0.155 | -.133 | 0.059 | .009 |
